# VUV/UV light inducing accelerated phenol degradation with a low electric input†
†Electronic supplementary information (ESI) available. See DOI: 10.1039/c6ra26043h



**DOI:** 10.1039/c6ra26043h

**Published:** 2017-01-23

**Authors:** Mengkai Li, Dong Wen, Zhimin Qiang, John Kiwi

**Affiliations:** a Key Laboratory of Drinking Water Science and Technology , Research Center for Eco-Environmental Sciences , University of Chinese Academy of Sciences , Chinese Academy of Sciences , 18 Shuang-qing Road , Beijing 100085 , China . Email: qiangz@rcees.ac.cn ; Tel: +86 10 62849632; b Ecole Polytechnique Fédérale de Lausanne , EPFL-SB-ISIC-GPAO , Station 6 , CH-1015 Lausanne , Switzerland . Email: john.kiwi@pfl.ch ; Email: john.kiwi@epfl.ch ; Tel: +41 21 6936150

## Abstract

This study presents the first evidence for the accelerated degradation of phenol by Fenton's reagent in a mini-fluidic VUV/UV photoreaction system (MVPS). A low-pressure mercury lamp used in the MVPS led to a complete degradation of phenol within 4–6 min. The HO˙ and HO_2_˙ originating from both Fenton's reagent and VUV photolysis of water were identified with suitable radical scavengers. The effects of initial concentrations of phenol, H_2_O_2_ and Fe^3+^ as well as solution pH on phenol degradation kinetics were examined. Increasing the initial phenol concentration slowed down the phenol degradation, whereas increasing the initial H_2_O_2_ or Fe^3+^ concentration accelerated the phenol degradation. The optimal solution pH was 3.7. At both 254 and 185 nm, increasing phenol concentration enhanced its absorption for the incident photons. The reaction mechanism for the degradation of phenol was suggested consistent with the results obtained. This study indicates that the VUV/UV photo-Fenton process has potential applications in the treatment of industrial wastewater containing phenol and related aromatic pollutants.

## Abstract 2

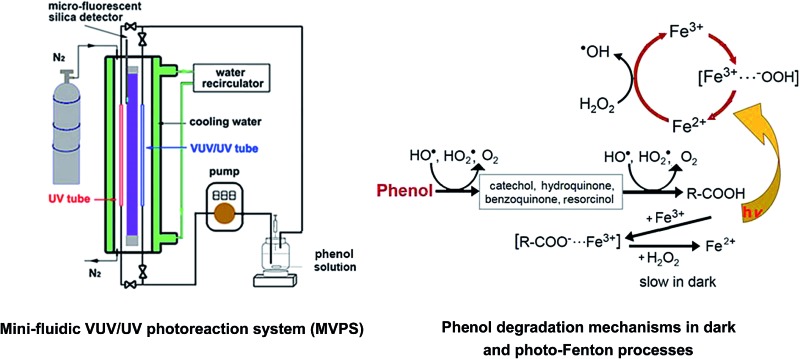
This study presents the kinetics and mechanism for the accelerated degradation of phenol in a mini-fluidic VUV/UV photoreaction system.

## Introduction

Industrial wastewater decontamination has been a research topic during the last few decades directed to abate toxic and recalcitrant pollutants of industrial origin.^1–3^ Phenol can only be degraded slowly by bacteria with low to moderate concentrations in mixtures of biodegradable and non-biodegradable pollutants in industrial wastewaters.^4–8^ Phenols, bi-phenols, chloro-phenols and nitro-phenols need at the present time a lower cost removal system working within an acceptable time than the biological and chemical methods currently available.^9,10^ Some advanced oxidation processes (AOPs) have used preferentially TiO_2_ as a photocatalyst for this purpose. TiO_2_ generates highly oxidative radicals under band-gap irradiation (band gap of 3.0–3.2 eV).^11–14^ Under UV or UV-Vis irradiation, TiO_2_ and doped-TiO_2_, either suspended in aqueous solutions or fixed on suitable substrates, have been applied to degrade phenol with relatively slow kinetics.^15–20^ Moreover, to increase the dispersion and light absorption of TiO_2_ and doped-TiO_2_, Raschig-rings have been used as supports to accelerate the phenol degradation kinetics.^21,22^

The degradation of phenol(s) by AOPs at current remains slow and inefficient in spite of recent improvements in reactor technology, lamp design and other engineering aspects. Fenton and photo-Fenton studies in homogeneous/heterogeneous systems leading to phenol degradation have increased in recent years. Esplugas *et al.* reported a time of *ca.* 10 min for phenol degradation.^23^ More recently, Zazo *et al.* reported a time of *ca.* 4 h for a complete degradation of phenol by Fenton's reagent.^24^ The authors also reported the phenol degradation intermediates: catechol, hydroquinone, *p*-benzoquinone and resorcinol, followed by short organic-acids branched or not before the final mineralization.^24^ Pontes *et al.* reported phenol degradation within 40 min in homogeneous solution,^25^ and by Friedrich *et al.* within 45 min.^26^ Heterogeneous Fenton and photo-Fenton using Fe–clinoptilolite led to a phenol reduction within 35 min,^27^ while clay pillared-Fe led to a phenol reduction within 120 min.^28^ Pillared montmorillonite Fe and clay/silica/zeolite reduced phenol within 60 and 120 min, respectively.^29,30^

The mechanism for the homogeneous catalysis of H_2_O_2_ leading to highly oxidative radicals in the dark has been reviewed.^31–33^ The fully hydrated Fe(H_2_O)_6_^3+^ complex was reported as a catalytic species in the Fenton system at pH < 3.^34^ At pH ≤ 3.5, the complexes Fe(OH)(H_2_O)_5_^2+^ and Fe(OH)_2_(H_2_O)_4_^+^ interact with H_2_O_2_ in the Fenton system, which has been comprehensively described.^35,36^ These Fe–aqua complexes have optical absorption up to 390 nm.^7,8^ The basic Fenton reactions are presented in eqn (1)–(4), whilst eqn (5) and (6) refer to the photo-Fenton reactions. Note that in eqn (4) and (5), Fe(OH)^2+^ represents a simplified form of Fe(OH)(H_2_O)_5_^2+^.

1 Fe^2+^ + H_2_O_2_ → Fe^3+^ + HO˙ + OH^–^, *k*_1_ = 76 M^–1^ s^–1^

2 Fe^3+^ + H_2_O_2_ → FeOOH^2+^ + H^+^, *k*_2_ = 2 × 10^–2^ M^–1^ s^–1^

3 FeOOH^2+^ → Fe^2+^ + HO_2_˙

4 Fe^3+^ + H_2_O → Fe(OH)^2+^ + H^+^

5 Fe(OH)^2+^ + *hν* → Fe^2+^ + HO˙

6 H_2_O_2_ + *hν* → 2HO˙

The photo-Fenton reactions presented in eqn (5) and (6) generate additional HO˙ through the light activated reduction of Fe(OH)^2+^ and the direct photolysis of H_2_O_2_. This accelerates the degradation of organic compounds compared to the dark Fenton system.^34^ The HO˙ generated led in the first reaction step to an aromatic ring addition to the phenol, leading to the hydroxyl-cyclohexadienyl radical.^37,38^

The objective of this study was to achieve an accelerated degradation of phenol by the Fenton's reagent (Fe^2+/3+^/H_2_O_2_) in a more advanced photo-reactor by way of VUV/UV irradiation. Due to a short degradation time and a low electric input, this VUV/UV photo-Fenton process only required a low energy consumption. The experiments were carried out in a mini-fluidic VUV/UV photoreaction system (MVPS), which provided VUV/UV or UV irradiation with an accurate quantification of UV fluence. The light absorption by each of the solution components in the photo-reactor was determined as well as the identity of intermediate oxidative radicals. The effects of initial phenol concentration, initial H_2_O_2_ concentration, and solution pH on phenol degradation kinetics were examined in detail. The reaction mechanism leading to phenol degradation was also suggested.

## Experimental section

### Chemical reagents and analytical methods

All solutions were prepared in ultrapure water produced from a Milli-Q system (Advantage A10, Millipore, USA). Chemicals used were of at least reagent grade and purchased from Sigma-Aldrich (St. Louis, MO, USA) or Thermo Fisher Scientific (Fair Lawn, NJ, USA). Fenton experiments were conducted employing FeCl_3_ and H_2_O_2_ (∼35% by weight). The initial pH was adjusted by NaOH and HCl solutions. 1,4-Benzoquinone (BQ) and *tert*-butanol (TBA) were used as O_2_˙^–^ and HO˙ scavengers, respectively. Uridine (0.12 mM) and methanol (0.10 mM) were utilized as chemical actinometers to determine the UV fluence rate output from the MVPS.

Phenol concentration was determined by using a high-performance liquid chromatograph coupled with a photodiode-array detector (HPLC/DAD, Agilent Technologies, US). Methanol concentration was determined by a gas chromatograph equipped with a flame ionization detector (GC/FID, Shimadzu, Japan). Uridine concentration was measured by light absorption at 664 nm on a UV-Vis spectrophotometer (2600, Shimadzu, Japan) directly linked to the MVPS. H_2_O_2_ concentration was measured by Titanium(iv) oxysulfate (TiOSO_4_) with a detection limit of 0.01 mg L^–1^.^39^ Total organic carbon (TOC) was analyzed with a TOC analyzer (TOC-VCPH, Shimadzu, Japan).

### VUV/UV and UV irradiations in the MVPS

To overcome the limitations of traditional batch VUV/UV photo-reactors, an innovative design of a bench-scale MVPS was developed in our previous study (see Fig. S1†).^39,40^ The photo-reactor chamber was cylindrical (length = 300 mm, outer diameter = 40 mm) with a jacketed quartz-wall. An 8 W low-pressure mercury lamp (arc length = 200 mm, Wanhua Co., Zhejiang, China) manufactured by synthetic quartz was housed in the center of the photo-reactor, which could emit both 185 (VUV) and 254 nm (UV) beams. Cooling water was recirculated through the outer chamber at a constant temperature to maintain stable VUV and UV outputs from the LP lamp. A synthetic quartz tube (*i.e.*, VUV/UV tube, high VUV transmittance) and a Ti-doped silica tube (*i.e.*, UV tube, no VUV transmittance) were installed parallel to the lamp in the photo-reactor chamber with the same distance (*ca.* 5 mm) to the lamp surface. The inner diameter and length of both tubes were 2 and 100 mm, respectively. The reaction solution flowing through the VUV/UV and UV tubes could receive the 185/254 nm and the 254 nm irradiations, respectively. Nearly same UV (at 254 nm) fluence rate (FR) could be obtained in the VUV/UV and UV tubes because both tubes had a very high 254 nm transmittance and were positioned at the same distance to the lamp surface. Nitrogen gas was flushed through the inner chamber of the photo-reactor, to eliminate the air absorption (primarily O_2_) of VUV and thus maximize the VUV light reaching the reaction solution. By using the chemical actinometers of uridine and methanol, the VUV and UV FRs (*i.e.*, *E*_o,VUV_ and *E*_o,UV_) were determined to be 1.7 and 14.5 mW cm^–2^, respectively. An UV-Vis spectrophotometer (2600, Shimadzu, Japan) was installed in the MVPS to provide online absorbance measurements.

### Experimental procedures and reduction equivalent exposure time

After a 15 min lamp equilibration period, the phenol solution of 50 mL was circulated in the MVPS by a peristaltic pump. Samples of 1 mL each were taken out at preselected times for chemical analysis. During the MVPS runs, the reaction solution was recirculated sequentially through the exposure section (*i.e.*, the VUV/UV or UV tube) and the dark section until a desired fluence was reached. The reduction equivalent exposure time (*t*_ree_, s) is defined as the total reaction time (*t*, s) multiplied by the ratio of the exposure volume of the operation tube (π*r* ^2^ *h*, m^3^) to the total sample volume (*V*, m^3^):7
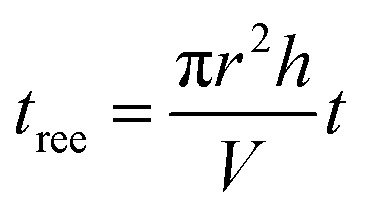
where *r* and *h* are the radius and the length of the VUV/UV (or UV) tube, respectively. In other words, *t*_ree_ represents the exposure time when the whole reaction solution receives the exposure under the same exposure conditions (*i.e.*, the same *E*_o,UV_ and *E*_o,VUV_) in the relevant tube. The VUV (*F*_VUV_, mJ cm^–2^) or UV (*F*_UV_, mJ cm^–2^) fluences can be estimated as follows:

8 *F*_VUV_ = *E*_o,VUV_*t*_ree_

9 *F*_UV_ = *E*_o,UV_*t*_ree_

## Results and discussion

### Phenol absorption spectrum and degradation kinetics and TOC reduction in the MVPS

**Fig. 1:**
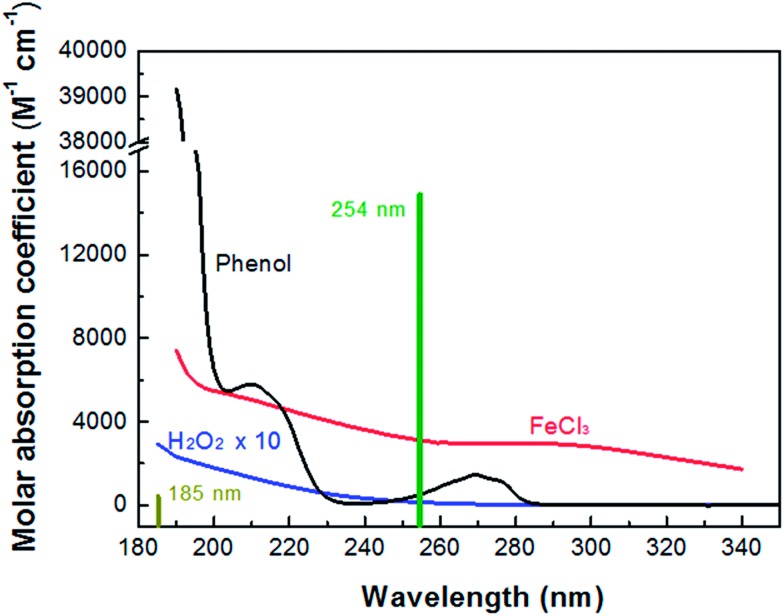
Absorption spectra of H_2_O_2_, FeCl_3_ and phenol and mercury resonance lines at 254 and 185 nm of the low-pressure mercury lamp.

Fig. 1 shows the absorption spectra of H_2_O_2_, FeCl_3_ and phenol. Note that the mercury resonance lines at 185/254 nm (*i.e.*, in the VUV/UV tube) or 254 nm (*i.e.*, in the UV tube) irradiations were used throughout this study. The molar absorption coefficients of H_2_O_2_, phenol and Fe(iii) increased towards shorter wavelengths. The light absorption of H_2_O_2_ was so low that it could not absorb a significant amount of VUV/UV and UV photons at the low concentrations (*i.e.*, mostly 0.147 mM) used in the MVPS. The Fe (d–d) transitions take place in the nanosecond time scale, and the intensity of these transitions giving rise to the Fe(iii)-spectrum depends on the counter-ion, pH and Fe(iii) concentration.^41,42^

**Fig. 2:**
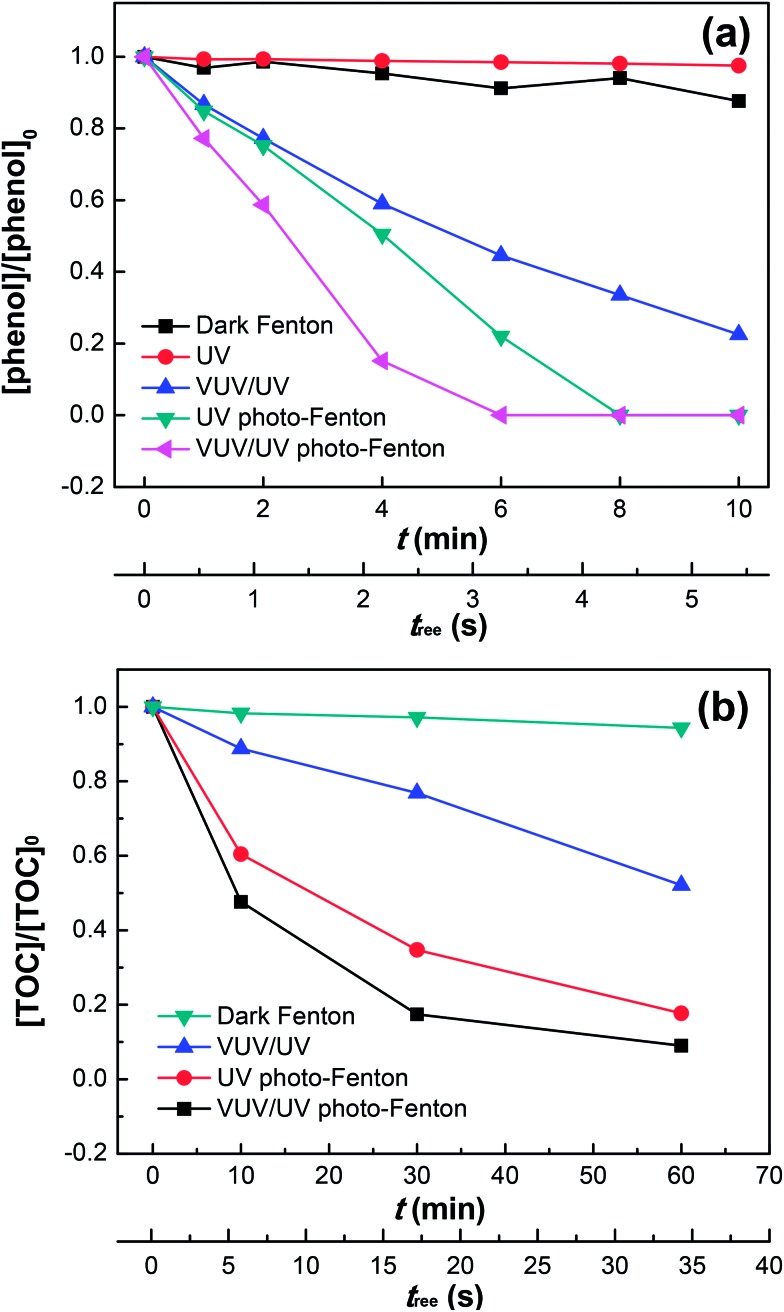
Phenol degradation (a) and TOC removal (b) in the dark Fenton, UV, VUV/UV, UV photo-Fenton and VUV/UV photo-Fenton processes at pH_0_ = 3.7. Experimental conditions: (a) [phenol]_0_ = 0.011 mM, [H_2_O_2_]_0_ = 0.147 mM, [Fe^3+^]_0_ = 0.05 mM; and (b) [phenol]_0_ = 0.055 mM, [H_2_O_2_]_0_ = 0.735 mM, [Fe^3+^]_0_ = 0.25 mM.

Fig. 2a shows the phenol degradation by the dark Fenton, UV or VUV/UV photolysis, UV photo-Fenton, and VUV/UV photo-Fenton processes at an initial pH of 3.7. The VUV/UV in the absence of the Fenton's reagent (Fe^2+/3+^/H_2_O_2_) induced phenol degradation due to the VUV (185 nm) light generating HO˙ from the photolysis of water.^43,44^ Without UV and VUV irradiation, little phenol degradation was found although both Fe^3+^ and H_2_O_2_ were present. UV and VUV/UV photo-Fenton processes led to a complete phenol degradation within 6–8 min. The photochemical behavior of the Fe(iii)–aqua complexes plays a determining role in pollutant degradation.^35,36^ At pH 3.7 in this study, Fe(H_2_O)_6_^3+^ was partially transformed into Fe(iii)(OH)(H_2_O)_5_^2+^, which was able to undergo ligand-to-metal-charge-transfer. This transformation depends on the excitation wavelength and involves inner sphere photo-induced electron transfer leading to the Fe(ii)–aqua complex (*i.e.*, Fe(ii)(H_2_O)_6_^2+^) and HO˙, as shown in eqn (5) above.

The HO˙ leading to phenol degradation was generated by the VUV (185 nm) photolysis of water and the Fe^3+^ photo-reduction. In addition, VUV also generates H˙ and e_aq_^–^ in aqueous solutions due to homolysis and water ionization.^43,44^ Fig. 2a shows that UV (254 nm) by itself did not lead to phenol degradation. The VUV (185 nm) photons preferentially react with water (concentration of 55.6 M) compared to phenol (0.011 mM). Hence, the VUV (185 nm) photon absorption by phenol only contributed marginally to the phenol degradation (see Table 1). When H_2_O_2_ and Fe^3+^ were added during phenol degradation under UV or VUV/UV irradiation, the higher efficiency of phenol degradation could be explained by the effective Fe(ii)/Fe(iii) inter-conversion and subsequently the higher generation of oxidative radicals. The Fe(OH)(H_2_O)_5_^2+^ may also react with dissolved oxygen in the reaction solution to form O_2_˙^–^, thus enhancing the phenol degradation. It has been reported that dissolved oxygen is able to enhance Fenton/photo-Fenton reactions.^37,38^

**Table 1:** Solution parameters determining the light absorption during phenol degradation in the MVPS^*a*^

Reactant	*C* (mM)	*ε*_254_ (M^–1^ cm^–1^)	*ε*_185_ (M^–1^ cm^–1^)	*A*_254_	*A*_185_	*P*_254_ (%)	*P*_185_ (%)
H_2_O	55 600	0.0002	0.03	0.01	1.8	5.7	68.1
Phenol	0.011	472	39 178	0.005	0.431	3.0	16.3
H_2_O_2_	0.147	19	341	0.003	0.050	1.6	1.9
FeCl_3_	0.05	3140	7250	0.157	0.363	89.7	13.7
Total				0.175	2.644	100	100

^*a*^ *C* is the molar concentration of each solution component; *ε*_254_ and *ε*_185_ are the molar absorption coefficient of each solution component at 254 and 185 nm, respectively; *A*_254_ and *A*_185_ are the absorbance of each solution component for 1 cm optical path length at 254 and 185 nm, respectively (*A*_254_ = *ε*_254_ × *C* and *A*_185_ = *ε*_185_ × *C*); *P*_254_ is the ratio of *A*_254_ of a specific solution component to the sum of *A*_254_ of all solution components (*i.e.*, total *A*_254_ = 0.175); and *P*_185_ is the ratio of *A*_185_ of a specific solution component to the sum of *A*_185_ for all solution components (*i.e.*, total *A*_185_ = 2.644).

Fig. 2b presents the mineralization of phenol under different irradiation conditions up to 60 min. Only 90% of the initial phenol was converted into CO_2_ in the VUV/UV photo-Fenton process. This suggests the existence of long-lived organic intermediates in the reaction solution, such as catechol, hydroquinone, *p*-benzoquinone and resorcinol as reported by Zazo *et al.*,^24^ which probably accounts for the tail after 30 min. Note that a higher concentration of phenol, H_2_O_2_ and Fe^3+^ was used here than that in Fig. 2a, because TOC could only be measured accurately at a higher phenol concentration (*i.e.*, 0.055 mM). The total mineralization reaction of phenol can be written as follows:

10 C_6_H_5_OH + 14H_2_O_2_ → 6CO_2_ + 17H_2_O

The fractions of the incident photons absorbed by each solution component (*i.e.*, Fe^3+^, H_2_O_2_, phenol, and H_2_O) during the TOC removal (Fig. 2b) is shown Fig. S2 (ESI†).

### Effects of initial phenol, H_2_O_2_ and Fe^3+^ concentrations on phenol degradation kinetics

**Fig. 3:**
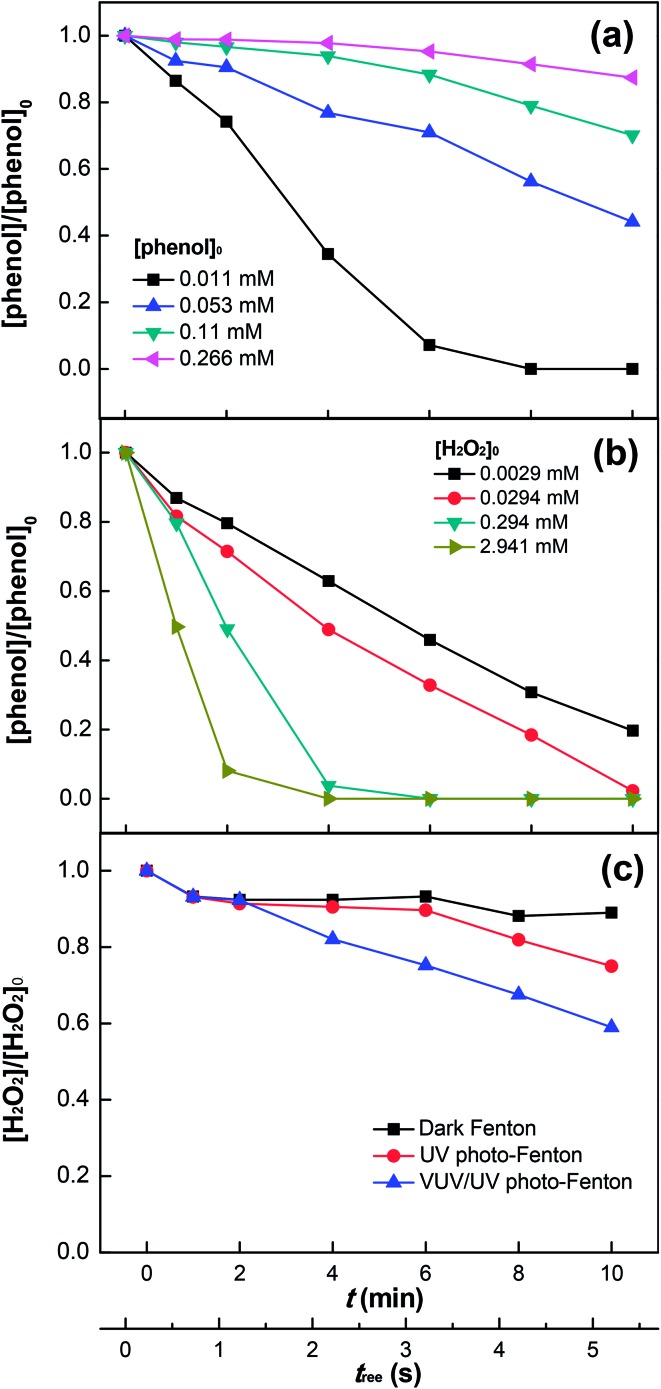
Effects of initial concentrations of phenol (a) and H_2_O_2_ (b) on phenol degradation as well as H_2_O_2_ decomposition (c) in the VUV/UV photo-Fenton process. Experimental conditions: [phenol]_0_ = 0.011 mM, [H_2_O_2_]_0_ = 0.147 mM, [Fe^3+^]_0_ = 0.05 mM, and pH_0_ = 3.7.

Fig. 3a shows phenol degradation as a function of the initial phenol concentration in the range of 0.011–0.266 mM. High concentrations of phenol decreased the degradation rate, due to the competition for the oxidative radicals in solution. In addition, the increasing phenol concentration would induce a higher fraction of UV (254 nm) photons absorbed by phenol, leaving less photons for the photo-reduction of Fe^3+^ (see eqn (5)). Similarly, this would also lower the amount of HO˙ generated by the VUV photolysis of water. Therefore, at high concentrations of phenol, the radicals generated by the Fenton's reagent (Fe^2+/3+^/H_2_O_2_) would not be enough to degrade effectively an increased amount of phenol. Although secondary reactions due to these oxidative radicals still led to the degradation of phenol, only a low efficiency was observed.^5,7,48^

Fig. 3b shows that the degradation rate of phenol increased with increasing H_2_O_2_ concentration, which implies that the interaction of H_2_O_2_ with phenol was significant. This can be explained by the photolysis of the intermediate complex/exciplex [Fe^3+^···phenol···H_2_O_2_] in solution.^23–27^ H_2_O_2_ could also scavenge HO˙ in solution,^11–14^ but this was not significant at H_2_O_2_ concentrations used in this study (*i.e.*, ≤2.941 mM). Hence, no inhibition of phenol degradation was seen in Fig. 3b. Moreover, the increasing Fe^3+^ concentration (up to 0.5 mM) could also enhance the phenol degradation (see Fig. S3, ESI†), which allowed Fe^3+^ to absorb more 254 and 185 nm photons (see eqn (5)), thus enhancing the HO˙ generation. Concomitantly, this would also decrease the fraction of photons absorbed by H_2_O_2_ and subsequently the HO˙ generation through H_2_O_2_ photolysis (see eqn (6)). Comparatively, the negative effect of the decreased H_2_O_2_ photolysis was less significant. The Cl^–^ ions added with FeCl_3_ could substitute water in the first coordination sphere of Fe(OH)(H_2_O)_5_^2+^ to yield mixed ligand complexes, such as FeCl(H_2_O)_5_^2+^ and FeCl_2_(H_2_O)_4_^+^.^41,45,46^

Fig. 3c shows the H_2_O_2_ consumption during phenol degradation. It is readily seen that using the Fenton's reagent in the dark led to a small H_2_O_2_ consumption due to the slow radical generation (*i.e.*, HO˙ and HO_2_˙) (see eqn (1) and (3)). A higher H_2_O_2_ consumption was observed when UV irradiation was applied. The reason is that more H_2_O_2_ was consumed by Fe^2+^, which was effectively photo-generated from Fe^3+^ (see eqn (4) and (5)), and by HO˙ in the radical scavenging process as well. Under VUV/UV irradiation, a significant increase in H_2_O_2_ consumption was observed compared to that under UV irradiation, because of the enhanced Fe^3+^ reduction, water photolysis,^43,44^ and H_2_O_2_ photolysis by the VUV light.

The VUV light generates radicals and ions in water as reported by previous researchers.^43,44^ VUV photolysis of water leads to both radicals HO˙, HO_2_˙, O_2_˙^–^, H˙ and stable species such as H_2_O_2_ and O_2_. In this study, the kinetics of phenol degradation depended on the absorbers in the UV and VUV regions (see Table 1). Even though the H_2_O_2_/UV process presents similarities with the VUV/UV process, there are substantial differences: (a) in the H_2_O_2_/UV process, the photolysis of H_2_O_2_ only generates HO˙; whereas in the VUV/UV process, the photolysis of water generates ˙OH, H˙ and e_aq_^–^ (H_2_O + *hν* (185 nm) → ˙OH + H^+^ + e_aq_^–^), which modifies the observed radical reactions.^43,44^ The direct addition of e_aq_^–^ to H_2_O_2_ during VUV irradiation (*i.e.*, H_2_O_2_ → HO_2_^–^ + H^+^) was not possible in the Marcus inverse region,^8^ because the activation energy necessary for this reaction was not available.

**Fig. 4:**
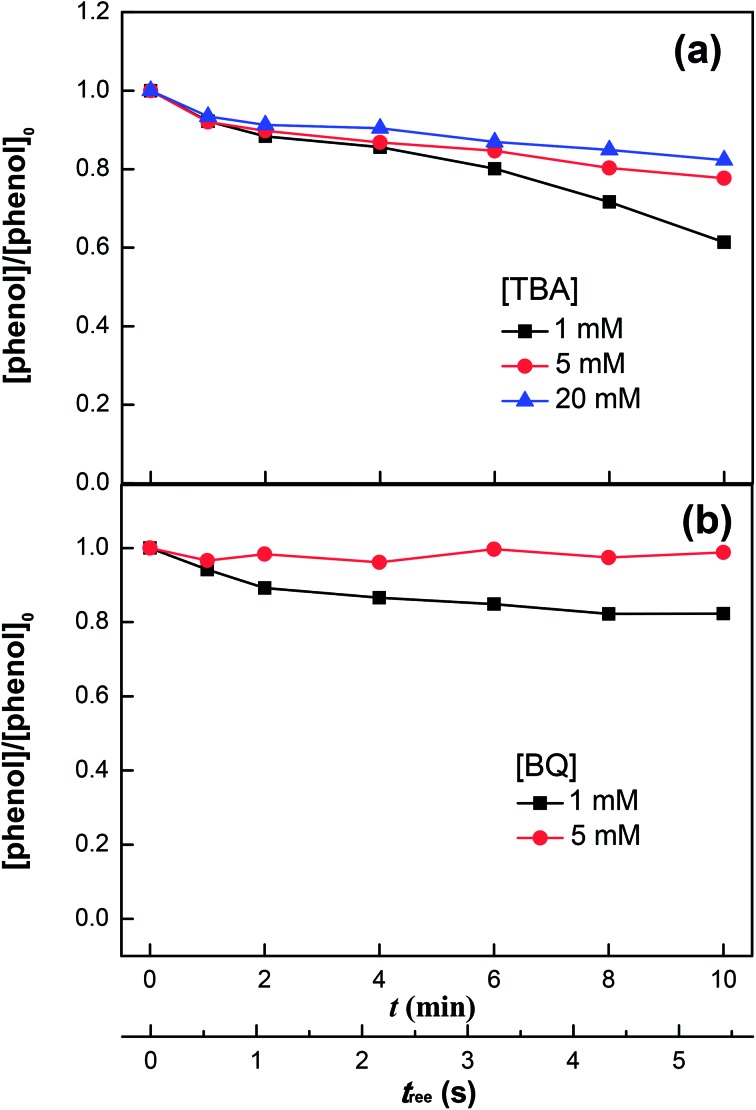
Phenol degradation in the VUV/UV photo-Fenton process in the presence of *tert*-butanol (TBA) (a) and benzoquinone (BQ) (b). Experimental conditions: [phenol]_0_ = 0.011 mM, [H_2_O_2_]_0_ = 0.147 mM, [Fe^3+^]_0_ = 0.05 mM, and pH_0_ = 3.7.

The decomposition of H_2_O_2_ produced the oxidative radicals HO˙ (in the first place) and HO_2_˙ (to a lesser degree), as demonstrated by the scavenging experiments with TBA and BQ (see Fig. 4). The decomposition of H_2_O_2_ involves eqn (1), (2) and (6) as well as additionally the reactions reported in literature:^31,37,47^

11 H_2_O_2_ + HO˙ → H_2_O + HO_2_˙

12 H_2_O_2_ + HO_2_˙ → H_2_O + O_2_ + HO˙

13 Fe^2+^ + HO_2_˙ → Fe^3+^ + HO_2_^–^

14 Fe^3+^ + HO_2_˙ → Fe^2+^ + H^+^ + O_2_

15 2HO˙ → H_2_O_2_

 Eqn (11) and (15) denote the scavenging and recombination of HO˙, respectively. As the H_2_O_2_ concentration increased, both reactions fostered the decomposition of H_2_O_2_.

### Identification of oxidative radicals produced in the VUV/UV photo-Fenton process

Fig. 4a shows the phenol degradation under VUV/UV irradiation with TBA used as a HO˙ scavenger. HO˙ is scavenged by TBA with a reaction rate of 1.9 × 10^9^ M^–1^ s^–1^ and reacts with phenol at a diffusion controlled reaction rate of 9.6 × 10^9^ M^–1^ s^–1^.^47^ It is readily seen that increasing the TBA concentration inhibited phenol degradation due to the quenching of the HO˙.

Fig. 4b shows the phenol degradation in the presence of BQ, an HO_2_˙ scavenger. The phenol degradation slowed down at higher BQ concentrations. BQ scavenges HO_2_˙ with a reaction rate of 9.6 × 10^8^ M^–1^ s^–1^ according to the reaction:

16 BQ + HO_2_˙ → BQ˙^–^ + H^+^ + O_2_

### Mechanism for phenol degradation by Fenton's reagent under VUV/UV irradiation

**Fig. 5:**
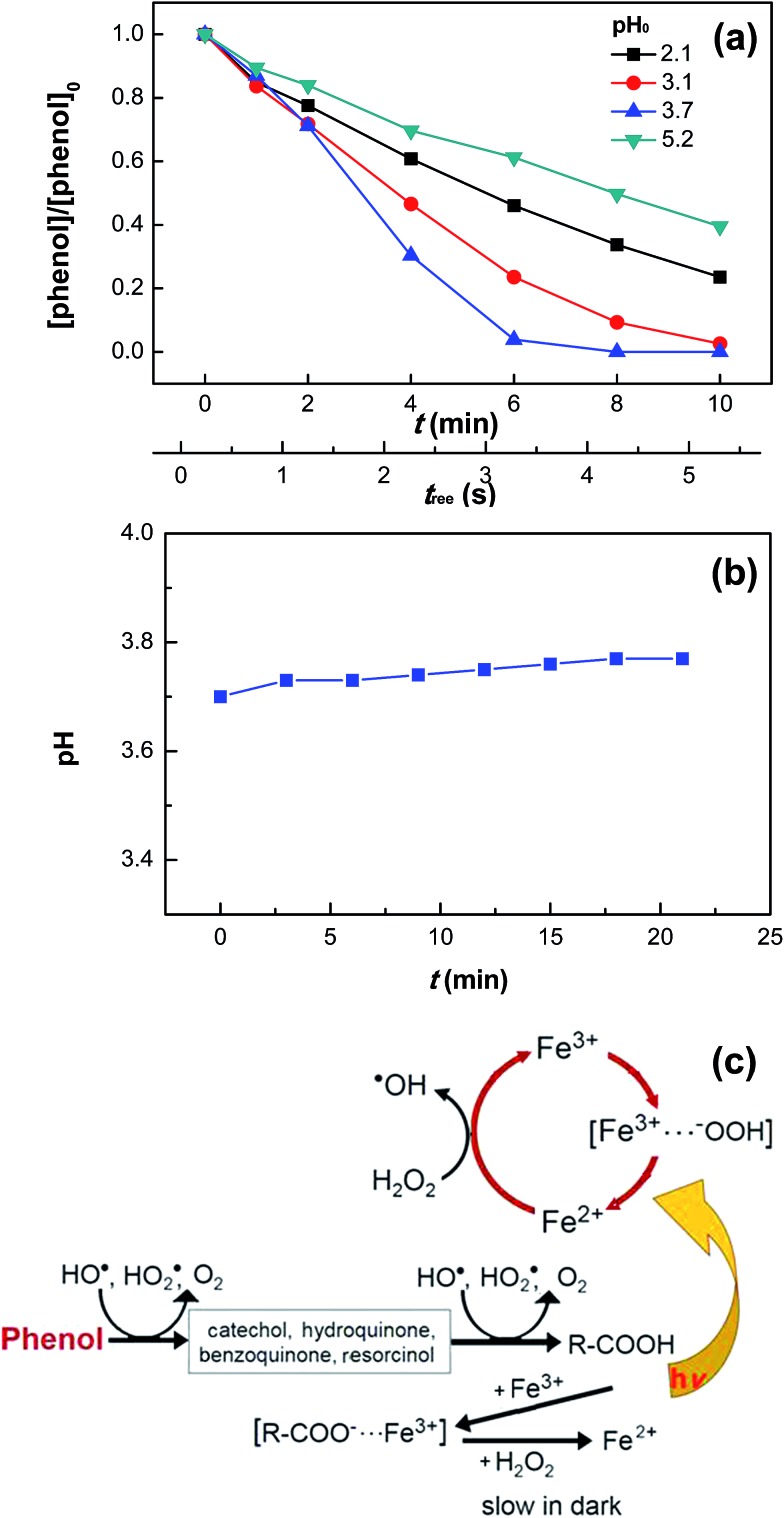
Phenol degradation at various initial pH values (a) and pH variation (pH_0_ = 3.7) (b) in the VUV/UV photo-Fenton process as well as proposed reaction mechanisms for the dark Fenton and VUV/UV photo-Fenton processes (c). Experimental conditions: [phenol]_0_ = 0.011 mM, [H_2_O_2_]_0_ = 0.147 mM, and [Fe^3+^]_0_ = 0.05 mM.

Fig. 5a shows the effect of initial pH on phenol degradation in the VUV/UV photo-Fenton process. Both Fe(OH)^2+^ reduction and H_2_O_2_ hydrolysis under UVC light (see eqn (5) and (6)), generated additional HO˙ compared to the dark Fenton system.^31,34,46^ The Fe(iii)–aqua complexes present different molar absorption coefficients and optical absorption wavelength ranges as a function of the solution pH.^35,36^ This in turn induced a different rate of phenol degradation and quantum yield as a function of pH. The highest phenol degradation rate was found at pH 3.7. At this pH, the initial fully coordinated Fe^3+^ ion under strongly acidic conditions, Fe(H_2_O)_6_^3+^, could incorporate an OH^–^ group in its structure leading to the formation of Fe(OH)(H_2_O)_5_^2+^.^35,36,42^ Under UVC light irradiation, Fe(OH)(H_2_O)_5_^2+^ led to the formation of HO˙ (see eqn (5)). The relatively slower phenol degradation rates at pHs 2.1 and 3.1 were probably due to the acidic Fe–aqua complexes substituted by Cl^–^ in the first coordination sphere such as FeCl(H_2_O)_5_^2+^ and FeCl_2_(H_2_O)_4_^2+^.^47^ At pH 5.2, the Fe–aqua complex, Fe(OH)_2_(H_2_O)_4_^+^, incorporated progressively one more OH^–^ group slowing down the phenol degradation rate.^48^ The degradation of other organic pollutants by photo-Fenton processes have been reported recently.^49,50^

Fig. 5b shows that during phenol degradation under VUV/UV irradiation, the solution pH moved slightly to more basic values. This suggests that the phenol degradation was mainly due to the HO˙/OH^–^ conversion. Fenton reactions induced the hydroxylation of the aromatic ring of phenol through the hydroxy-cyclohexadienyl radical by the addition of HO˙, which subsequently led to the formation of quinones and other intermediates shown in Fig. 5c and also found during phenol degradation by the Fenton's reagent by Zazo *et al.* ^24^ A mechanistic cycle for phenol degradation in the dark and by a photo-Fenton system under UVC irradiation is suggested in Fig. 5c.

### Light absorption by each solution component during phenol degradation with various initial concentrations

**Fig. 6:**
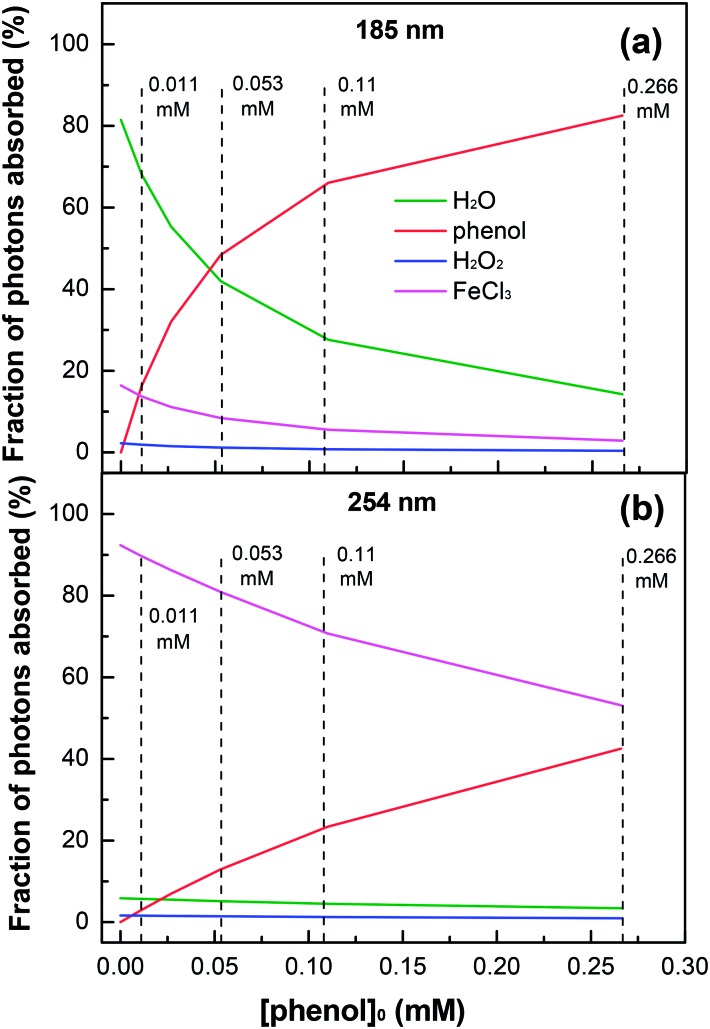
Fractions of 185 (a) and 254 nm (b) photons absorbed by each solution component as a function of initial phenol concentration in the VUV/UV photo-Fenton process. Experimental conditions: [H_2_O_2_]_0_ = 0.735 mM, [Fe^3+^]_0_ = 0.25 mM, and pH_0_ = 3.7.

Although the effect of initial phenol concentration on its degradation kinetics was already examined in Fig. 3a, the specific contribution of the highly oxidative radical species leading to phenol degradation has not been completely elucidated. This remains a controversial issue. Fig. 6a shows the fraction variation of the 185 nm photons absorbed by each solution component as a function of the phenol concentration. For the degradation of low-concentration phenol (*i.e.*, [phenol]_0_ = 0.011 mM) by the VUV/UV photo-Fenton process, the fraction of 185 nm photons absorbed by water was 68.1%, while those by phenol and FeCl_3_ were 16.3% and 13.7%, respectively, indicating that the effect of the additional VUV irradiation was the HO˙ generation from VUV photolysis of water as compared to that of the UV photo-Fenton process. The enhancement for Fe^3+^ reduction and direct photolysis of phenol by the VUV (185 nm) light was rather small. When the phenol concentration increased to 0.266 mM, phenol could absorb 82.5% of the 185 nm photons. In this case, the HO˙ generation through VUV photolysis of water became much less important.

Fig. 6b shows the fraction of the 254 nm photons absorbed by each component in the solution as a function of phenol concentration. Because of the low molar absorption coefficient of water at 254 nm (see Table 1), the photon absorption by water was not significant. For a low phenol concentration, Fe^3+^ could absorb most of the 254 nm photons. However, when the phenol concentration increased from 0.011 to 0.266 mM, the fraction of the 254 nm photons absorbed by phenol increased from 3.0% to 42.5%, which considerably decreased the generation of HO˙ through eqn (4)–(6).

The results of photon absorption by the components in solution show that under UV and VUV irradiations, the 254 and 185 nm photons absorbed by phenol itself played an important role in phenol degradation, especially when the phenol concentration was high. The ionization of phenol requires 8.50 eV and may go through either a mono-photonic or bi-photonic absorption process. The 185 nm light is made up of 6.70 eV photons; therefore, it might induce a mono-photonic ionization of phenol. For the 254 nm photons (4.87 eV), a bi-photonic ionization of phenol was likely to occur. The ionization reactions are presented in eqn (17)–(19). These results demonstrate that the phenol concentration strongly affected its degradation kinetics and the VUV/UV photo-Fenton process was most suitable for the treatment of dilute phenol solutions.

17 Phenol + *hν* → phenol^+^ + e_aq_^–^

18 Phenol + e_aq_^–^ → phenol^–^

19 e_aq_^–^ + O_2_ + H^+^ → HO_2_˙

## Conclusions

This study addressed the optimization of the solution parameters leading to the enhanced abatement of phenol in a mini-fluidic VUV/UV photoreaction system. The phenol degradation required a reduced consumption of electricity because the VUV light was made full use. By the VUV/UV mercury resonant lines (185/254 nm), water and H_2_O_2_ generated the highly oxidative radicals (HO˙ and HO_2_˙) leading to phenol degradation. The generation of these oxidative radicals was further increased in the presence of Fe^3+^ ions in the photo-Fenton processes. The oxidative radicals which intervened in the phenol degradation were identified. In addition, the fraction of the incident photons absorbed by each solution component was quantified. For a dilute phenol solution (0.011 mM), the fraction absorbed by phenol was 16.3% for the VUV photons (185 nm) and 3.0% for the UV photons (254 nm), while for a high-concentration phenol solution (0.266 mM), the fraction increased to 82.5% (185 nm photons) and to 42.5% (254 nm photons). Due to a relatively low electrical energy used to generate the oxidative radicals to abate phenol, the VUV/UV photo-Fenton process has potential applications in the treatment of industrial wastewater containing phenol and related aromatic pollutants.

## References

[cit1] Malato S., Fernández-Ibáñez P., Maldonado M. I., Blanco J., Gernjak W. (2009). Catal. Today.

[cit2] Hoffmann M. R., Martin S. T., Choi W. Y., Bahnemann D. W. (1995). Chem. Rev..

[cit3] Roques H., Chemical Water Treatment: Principles and Practice, VCH Publishers Inc., New York, NY, 1996.

[cit4] Cheremissinoff N. P., Handbook of Water and Waste Water Treatment Technologies, Butterworth-Heinemann, Woburn, MA, 2002.

[cit5] Scott J. P., Ollis D. F. (1995). Environ. Prog..

[cit6] Legrini O., Oliveros E., Braun A. M. (1993). Chem. Rev..

[cit7] Aquatic and Surface Photochemistry, ed. G. R. Helz, R. G. Sepp and D. G. Crosby, Lewis Publishers, Boca Raton, FL, 1994.

[cit8] Stumm W. and Morgan J. J., Aquatic Chemistry: Chemical Equilibria and Rates in Natural Waters, John Wiley & Sons, Inc., New York, NY, 3rd edn, 1996.

[cit9] Machulek Jr A., Oliveira S. C., Osugi M. E., Ferreira V. S., Quina F. H., Dantas R. F., Oliveira S. L., Casagrande G. A., Anaissi F. J., Silva V. O., Cavalcante R. P., Gozzi F., Ramos D. D., da Rosa A. P. P., Santos A. P. F., de Castro D. C. and Nogueira J. A., Application of Different Advanced Oxidation Processes for the Degradation of Organic Pollutants, InTech, Sao Paulo, Brazil, 2013.

[cit10] Stefan M., Advanced Oxidation Processes for Water Treatment: Fundamental and Applications, IWA Publishing, Alliance House, London UK, 2017, in press.

[cit11] Fujishima A., Zhang X. T., Tryk D. A. (2008). Surf. Sci. Rep..

[cit12] Pelaez M., Nolan N. T., Pillai S. C., Seery M. K., Falaras P., Kontos A. G., Dunlop P. S. M., Hamilton J. W. J., Byrne J. A., O'Shea K., Entezari M. H., Dionysiou D. D. (2012). Appl. Catal., B.

[cit13] Banerjee S., Dionysiou D. D., Pillai S. C. (2015). Appl. Catal., B.

[cit14] Schneider J., Matsuoka M., Takeuchi M., Zhang J. L., Horiuchi Y., Anpo M., Bahnemann D. W. (2014). Chem. Rev..

[cit15] Matthews R. W. (1988). J. Catal..

[cit16] Environmental Health and Toxicology; https://sis.nlm.nih.gov, National Library of Medicine, USA, 2005.

[cit17] Balasubramanian G., Dionysiou D. D., Suidan M. T., Subramanian Y., Baudin I., Laine J. M. (2003). J. Mater. Sci..

[cit18] Wiesner M. R., Lowry G. V., Alvarez P., Dionysiou D., Biswas P. (2006). Environ. Sci. Technol..

[cit19] Rosas J. M., Vicente F., Santos A., Romero A. (2013). Chem. Eng. J..

[cit20] Veréb G., Manczinger L., Oszkó A., Sienkiewicz A., Forró L., Mogyorósi K., Dombi A., Hernádi K. (2013). Appl. Catal., B.

[cit21] Raja P., Bensimon M., Kulik A., Foschia R., Laub D., Albers P., Renganathan R., Kiwi J. (2005). J. Mol. Catal. A: Chem..

[cit22] Sampaio M. J., Silva C. G., Silva A. M. T., Vilar V. J. P., Boaventura R. A. R., Faria J. L. (2013). Chem. Eng. J..

[cit23] Esplugas S., Giménez J., Contreras S., Pascual E., Rodríguez M. (2002). Water Res..

[cit24] Zazo J. A., Casas J. A., Mohedano A. F., Gilarranz M. A., Rodríguez J. J. (2005). Environ. Sci. Technol..

[cit25] Pontes R. F. F., Moraes J. E. F., Machulek Jr A., Pinto J. M. (2010). J. Hazard. Mater..

[cit26] Friedrich L. C., Zanta C. L. P. S., Machulek Jr A., Silva V. D., Quina F. H. (2012). Sci. Agric..

[cit27] Bayat M., Sohrabi M., Royaee S. J. (2012). J. Ind. Eng. Chem..

[cit28] Catrinescu C., Arsene D., Apopei P., Teodosiu C. (2012). Appl. Clay Sci..

[cit29] Ayodele O. B., Lim J. K., Hameed B. H. (2012). Appl. Catal., A.

[cit30] Navalon S., Alvaro M., Garcia H. (2010). Appl. Catal., B.

[cit31] Sychiov A. Y., Isac V. G. (1995). Usp. Khim..

[cit32] Cieśla P., Kocot P., Mytych P., Stasicka Z. (2004). J. Mol. Catal. A: Chem..

[cit33] Sima J., Makanova J. (1997). Coord. Chem. Rev..

[cit34] Ruppert G., Bauer R., Heisler G. (1993). J. Photochem. Photobiol., A.

[cit35] Safarzadeh-Amiri A., Bolton J. R., Cater S. R. (1996). Sol. Energy.

[cit36] Wang D., Oppenländer T., El-Din M. G., Bolton J. R. (2010). Photochem. Photobiol..

[cit37] Walling C. (1975). Acc. Chem. Res..

[cit38] Ito S., Mitarai A., Hikino K., Hirama M., Sasaki K. (1992). J. Org. Chem..

[cit39] Li M. K., Qiang Z. M., Hou P., Bolton J. R., Qu J. H., Li P., Wang C. (2016). Environ. Sci. Technol..

[cit40] Li M. K., Qiang Z. M., Pulgarin C., Kiwi J. (2016). Appl. Catal., B.

[cit41] Kwan W. P., Voelker B. M. (2003). Environ. Sci. Technol..

[cit42] Balzani V. and Carassiti V., Photochemistry of Coordination Compounds, Academic Press, London, 1970.

[cit43] Robl S., Worner M., Maier D., Braun A. M. (2012). Photochem. Photobiol. Sci..

[cit44] Bagheri M., Mohseni M. (2015). J. Hazard. Mater..

[cit45] Inorganic Chemistry: Towards the 21st Century, ACS Symposium Series, ed. M. H. Chisholm, American Chemical Society, Washington, DC, 1983, vol. 211.

[cit46] Nadtochenko V., Kiwi J. (1998). Environ. Sci. Technol..

[cit47] Wardman P. (1989). J. Phys. Chem. Ref. Data.

[cit48] Dorfman L. M. and Adams G. E., National Standard Reference Data System, National Bureau of Standards 46, Washington, DC, 1973.

[cit49] Yang X. L., Chen W., Huang J. F., Zhou Y., Zhu Y. H., Li C. Z. (2015). Sci. Rep..

[cit50] Shi W., Du D., Shen B., Cui C. F., Lu L. J., Wang L. Z., Zhang J. L. (2016). ACS Appl. Mater. Interfaces.

