# Supplementary material for: VUV/UV light inducing accelerated phenol degradation with a low electric input
Source: RSC Adv. 2017 Jan 23;7(13):7640–7. doi: 10.1039/c6ra26043h (PMC5361170; doi:10.1039/c6ra26043h)
Supplement: Supplementary file 1 [file RA-007-C6RA26043H-s001.pdf]

Electronic Supplementary Material (ESI) for RSC Advances.
This journal is © The Royal Society of Chemistry 2017

Supplementary material

VUV/UV light inducing accelerated phenol degradation with a low electric input

Mengkai Li [a,b], Dong Wen [a], Zhimin Qiang [a,*] and John Kiwi [b,*]

[a] *Key Laboratory of Drinking Water Science and Technology, Research Center for Eco-Environmental Sciences, University of Chinese Academy of Sciences, Chinese Academy of Sciences, 18 Shuang-qing Road, Beijing 100085, China. E-mail: qiangz@rcees.ac.cn*

[b] *Ecole Polytechnique Fédérale de Lausanne, EPFL-SB-ISIC-GPAO, Station 6, CH-1015 Lausanne, Switzerland. E-mail: john.kiwi@pfl.ch*

* Corresponding authors.

E-mail: qiangz@rcees.ac.cn, Tel.: +86 10 62849632

E-mail: john.kiwi@epfl.ch, Tel.: +41 21 6936150

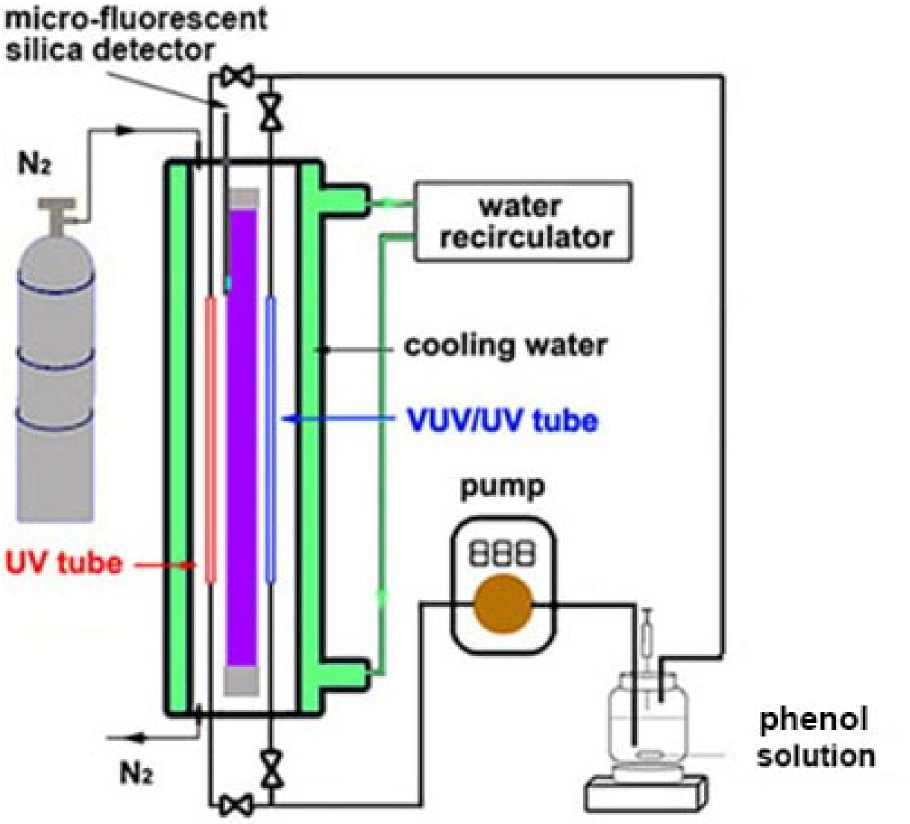

Fig. S1. Schematic diagram of the mini-fluidic VUV/UV photoreaction system (MVPS).[1]

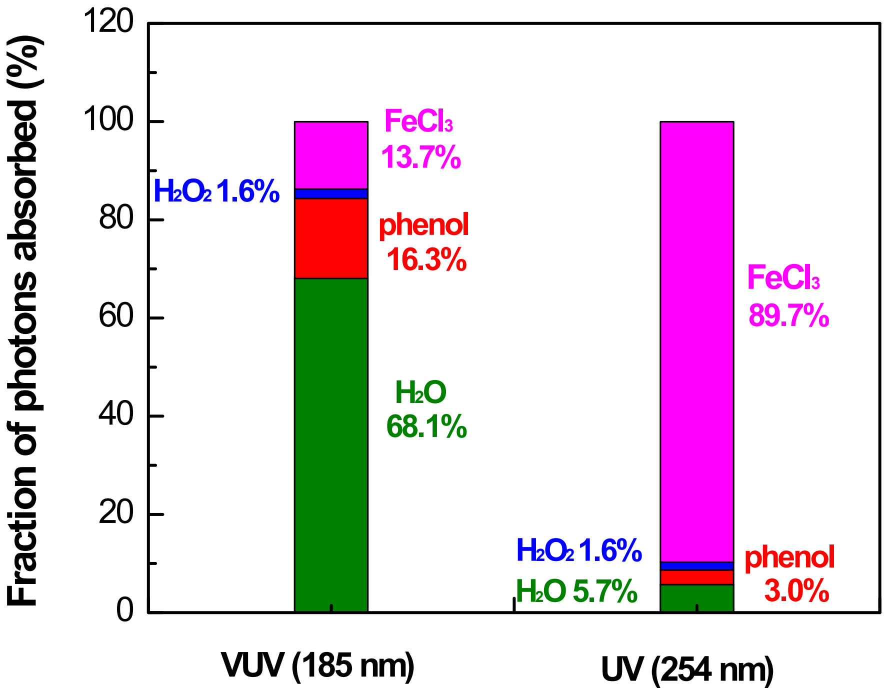

Fig. S2. Fractions of the photons absorbed by each solution component in the VUV/UV photo-Fenton process. Conditions: $[\text{phenol}]_0$ = 0.055 mM, $[H_2O_2]_0$ = 0.735 mM, $[Fe^{3+}]_0$ = 0.25 mM, and pH_0 = 3.7.

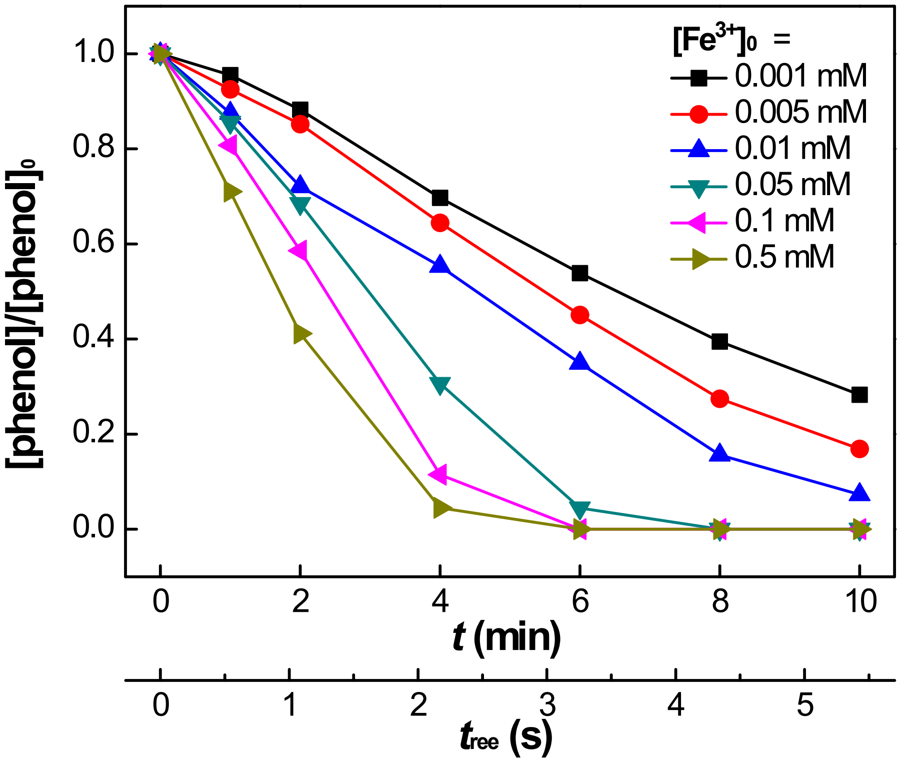

Fig. S3. Phenol degradation by the VUV/UV photo-Fenton process at various initial Fe^{3+} concentrations. Conditions: $[phenol]_0$ = 0.011 mM, $[H_2O_2]_0$ = 0.147 mM, and pH_0 = 3.7.

Reference

1 M. K. Li, Z. M. Qiang, P. Hou, J. R. Bolton, J. H. Qu, P. Li, C. Wang, *Environ. Sci. Technol.* 2016, **50**, 5849–5856.
